# Artery of Percheron infarct with multiple cranial nerve palsies and Horner Syndrome

**DOI:** 10.1016/j.ajoc.2025.102458

**Published:** 2025-10-14

**Authors:** William A. Sanfelippo, Matthew Oley, Hannah Harrelson, Nancy Vilar

**Affiliations:** University of Virginia Department of Ophthalmology, 1215 Lee Street, Box 800654, Charlottesville, VA, 22908-0816, USA

**Keywords:** Percheron, Horner syndrome

## Abstract

**Purpose:**

To report a case of a rare stroke variant involving the artery of Percheron leading to ocular motility compromise.

**Observations:**

A 65-year-old man presented to the neuro-ophthalmology clinic with multiple cranial nerve palsies and Horner Syndrome. He was three weeks post-thrombolytic therapy to treat an ischemic stroke, and his pertinent medical history consisted of hyperlipidemia and macular degeneration. On physical exam, the patient experienced bilateral vertical gaze paresis, incomplete left Horner syndrome, right-sided cranial nerve VI palsy, and partial left-sided cranial nerve VII palsy. Based on the infarction patterns demonstrated on MRI, the patient was diagnosed with an artery of Percheron infarct.

**Conclusions and importance:**

The artery of Percheron is a rare variant of posterior cerebral circulation that supplies both paramedian thalamic zones in addition to variably supplying the midbrain. Overall, this case highlights the importance of considering rare anatomical variants when working up ophthalmic deficits in a stroke patient. Early diagnosis can lead to adequate therapy and prevent complications in the future.

## 1 Introduction

The artery of Percheron is a rare variant of posterior cerebral circulation that supplies both paramedian thalamic zones in addition to variably supplying the midbrain. Infarction of the artery of Percheron is exceedingly rare, accounting for less than 2% of all thalamic strokes and can present with multiple different symptoms.^1^^,^^2^ We present the case of a 65-year-old man with history of hyperlipidemia and macular degeneration who presents to neuro-ophthalmology clinic with multiple cranial nerve palsies and Horner Syndrome, three weeks after ischemic infarct of the thalamus and midbrain. This case represents a unique presentation of an artery of Percheron infarct not previously described in the literature to the best of our knowledge.

## 2 Case report

The patient originally presented to an outside hospital after acutely suffering double vision, weakness of his left side, and then losing consciousness. At the outside facility he was treated with the thrombolytic tenecteplase and his mental status improved to baseline. At the time of discharge, he was noted to have right hypertropia on primary gaze, incomplete fascicular right oculomotor palsy, and bilateral complete downgaze/partial upgaze paresis. Six days after suffering a stroke, the patient was seen in the ophthalmology resident clinic where he was noted to have left incomplete Horner syndrome with positive apraclonidine test marked by ptosis and anisocoria, which was not previously described. This finding was in addition to an inability to supraduct or infraduct bilaterally and a right eye lateral gaze palsy. Three weeks following the patient's initial infarction, he was seen in the neuro-ophthalmology clinic. In addition to the findings previously noted, in the neuro-ophthalmology clinic the patient was observed to require a 16 PD base-down prism of the right eye and 6 PD base-out prism of the left eye. Cranial nerve V was intact in all divisions bilaterally. A partial cranial nerve VII palsy was noted on the left side with no lagophthalmos. In total the patient experienced bilateral vertical gaze paresis, incomplete left Horner syndrome, right-sided cranial nerve VI palsy, and partial left-sided cranial nerve VII palsy.

**Fig. 1:**
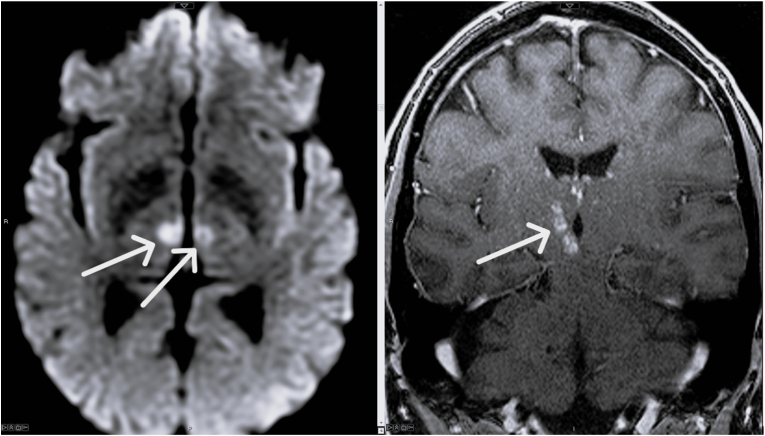
Left: axial diffusion weighted image showing hyperintense signal (diffusion restriction) within the bilateral medial thalami (right greater than left). Right: coronal T1 postcontrast image showing enhancing subacute infarct within the area of right sided infarct extending from the midbrain to the thalamus.

**Fig. 2:**
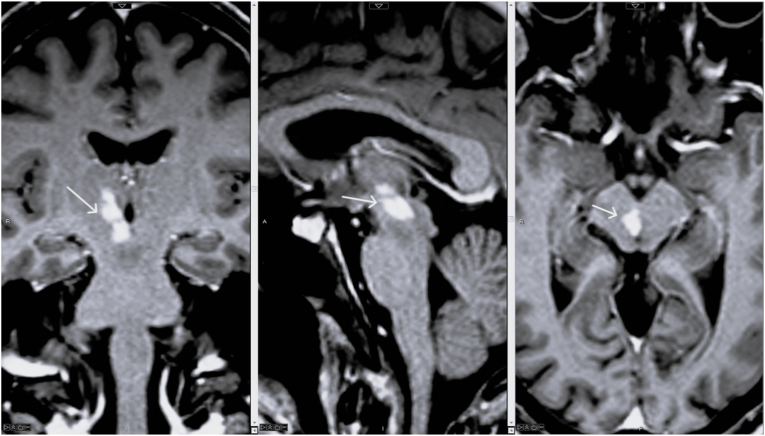
T1 postcontrast images in coronal, sagittal, and axial planes showing the enhancing subacute right infarct. Structures affected within the midbrain include the red nucleus, midbrain MLF, and periaqueductal gray matter.

MRI of the brain revealed an infarct in the left thalamus, infarct along the anteromedial right thalamus, and infarct of the right medial midbrain (Fig. 1). Structures affected within the midbrain include the red nucleus, midbrain medial longitudinal fasciculus (MLF), and periaqueductal gray matter (Fig. 2). MRA did not show evidence of flow-limiting stenosis or large vessel occlusion in the intracranial vasculature or any acute abnormality in the cervical vasculature. Based on the findings of the infarction pattern, the patient was diagnosed with an infarct of the artery of Percheron. On follow up MRI, the patient was also found to have findings involving bilateral cerebral peduncles consistent with ischemia/infarction.

## 3 Discussion

The thalamus and midbrain perforator blood supply has classically been divided into four separate subtypes. Type I and the most common variant involves the perforating branches individually arising from the right and left posterior cerebral arteries.^1^ For the Type IIa variant, both paramedian arteries supplying the thalamus originate from the P1 segment of a single posterior cerebral artery.^1^ The Type IIb variant of perforator blood supply involves the artery of Percheron arising from the P1 segment of a posterior communicating artery directing blood supply to both paramedian thalami and the rostral midbrain.^1^^,^^2^ In the Type III variant, an arcade gives off small perforating vessels from one arterial arc connecting the right and left posterior cerebral arteries.^1^ The artery of Percheron variant occurs in roughly 4%–12% of the population and infarcts of the variant account for 0.1%–2% of all ischemic strokes, making this type of stroke extremely rare.^1^ Classically, the symptoms of an artery of Percheron infarct are broad, but may include drowsiness progressing to coma, vertical gaze palsy, cognitive impairment, aphasia, dysarthria, motor deficits, and cerebellar signs.^1^^,^^2^ MRI with FLAIR and DWI is the imaging modality of choice to work up artery of Percheron infarcts as CTA and conventional angiography are unable to reliably show occlusions in arteries of such small diameter.^1^^,^^3^ Due to the wide variety in symptomatology and difficulty obtaining timely MRI, artery of Percheron infarcts may often be misdiagnosed or missed within the window when thrombolytic intervention may be beneficial.

This case sheds light on how thalamic and brainstem strokes lead to motility deficits along with other clinically apparent signs to the ophthalmologist. The patient's bilateral gaze paresis may be explained by his midbrain lesion affecting the vertical gaze center. As can be appreciated on MRI, the patient's ischemic infarct affects the medial longitudinal fasciculus, periaqueductal gray matter, along with the interstitial nucleus of Cajal. The patient's Horner syndrome is secondary to a lesion of the first-order neuron in the course of the descent through the midbrain.^4^ The patient's cranial nerve seven palsy may be explained by the ischemia appreciated in the cerebral peduncles affecting the cortical fibers of the seventh cranial nerve as they course from the primary motor cortex to the seventh nucleus in the pons. The patient's apparent cranial nerve six palsy is more difficult to explain by the appreciated ischemic regions on imaging. The nucleus of cranial nerve six is unaffected in the pons. Two hypotheses may explain the patient's right lateral gaze deficit. Intracranial pressure is known to increase following ischemic strokes including following mild to moderate strokes that may not cause significant edema.^5^ Following the patient's ischemic stroke and subsequent fluctuation in intracranial pressure, he suffered a cranial nerve six palsy. This hypothesis is further supported by the patient's recovery of lateral gaze on later follow up. A second possible explanation for the patient's horizontal gaze deficit is a rare outcome of thalamic infarcts, a pseudoabducens palsy. Pseudoabducens palsy has been reported as a rare manifestation of thalamic stroke with varying proposed mechanisms underlying the palsy from divergence paralysis to disruption of the inhibitory convergence pathways present in the thalamus.^6^

Small vessel disease is the most common cause of bilateral thalamic strokes, with an embolic source being less common.^5^ MRI findings in this patient are consistent with the etiology of small vessel disease, showing numerous T2/FLAIR deep white matter hyperintensities, favored to represent chronic ischemic microangiopathy. The patient's initial transthoracic echocardiogram was reassuring for any source of embolism; however, the patient later underwent a transesophageal echocardiogram which demonstrated at least a moderate-sized patent foramen ovale (PFO) with mobile interatrial septum and a “septal pouch”. The findings were considered high risk for recurrent neurologic events, and the patient underwent PFO closure for secondary prevention of cardioembolic events. Overall, the prognosis for artery of Percheron infarcts depends on the structures affected. Patients with bilateral paramedian thalamic infarcts without midbrain involvement tend to have better long-term outcomes than patients with midbrain involvement.^7^ Our patient was successfully treated with thrombolysis and saw immense initial recovery, progressing from coma-like state to baseline mental status within one day of treatment. However, the patient's visual symptoms remained three weeks following his initial infarct. Based on the patient's midbrain involvement, he is less likely to have a favorable long-term outcome, although based on his rapid treatment with thrombolysis he has greater potential for recovery. The patient was counseled on the importance of controlling risk factors for stroke including blood glucose, blood pressure, and cholesterol levels in addition to following closely with his primary care physician. He was advised to patch one eye to avoid diplopia as needed and scheduled for a three-month follow-up visit to re-check motility.

This case represents a previously unknown presentation of an artery of Percheron infarct. Artery of Percheron infarcts can be difficult to diagnose based on variant symptoms; however, rapid evaluation of symptoms including multiple ophthalmic manifestations and mental status changes is essential for timely treatment with thrombolysis. Awareness of artery of Percheron infarction in the differential of any ophthalmologist can prove beneficial to patients.

## CRediT authorship contribution statement

**William A. Sanfelippo:** Writing – review & editing, Writing – original draft, Data curation, Conceptualization. **Matthew Oley:** Writing – review & editing. **Hannah Harrelson:** Writing – review & editing. **Nancy Vilar:** Writing – review & editing, Supervision.

## Patient consent

Written consent to publish this case has not been obtained. This report does not contain any personal identifying information.

## Authorship

All authors attest that they meet the current ICMJE criteria for Authorship.

## Declaration of competing interest

The authors declare that they have no known competing financial interests or personal relationships that could have appeared to influence the work reported in this paper.
